# Autoencoder-Based Target Detection in Automotive MIMO FMCW Radar System

**DOI:** 10.3390/s22155552

**Published:** 2022-07-25

**Authors:** Sung-wook Kang, Min-ho Jang, Seongwook Lee

**Affiliations:** School of Electronics and Information Engineering, College of Engineering, Korea Aerospace University, Goyang-si 10540, Gyeonggi-do, Korea; sys77750@kau.kr (S.-w.K.); jmh17360@kau.kr (M.-h.J.)

**Keywords:** autoencoder, constant false alarm rate, frequency-modulated continuous wave radar, multiple-input and multiple-output, target detection

## Abstract

In general, a constant false alarm rate algorithm (CFAR) is widely used to automatically detect targets in an automotive frequency-modulated continuous wave (FMCW) radar system. However, if the number of guard cells, the number of training cells, and the probability of false alarm are set improperly in the conventional CFAR algorithm, the target detection performance is severely degraded. Therefore, we propose a method using a convolutional neural network-based autoencoder (AE) to replace the CFAR algorithm in the multiple-input and multiple-output FMCW radar system. In the AE, the entire detection result is compressed at the encoder side, and only significant signal components are recovered on the decoder side. In this work, by changing the number of hidden layers and the number of filters in each layer, the structure of the AE showing a high signal-to-noise ratio in the target detection result is determined. To evaluate the performance of the proposed method, the AE-based target detection result is compared with the target detection results of conventional CFAR algorithms. As a result of calculating the correlation coefficient with the data marked with the actual target position, the proposed AE-based target detection shows the highest similarity with a correlation of 0.73 or higher.

## 1. Introduction

Along with the rapid development of autonomous driving technology, the development of sensors for vehicles, such as cameras, lidars, and radars, has also accelerated. Among these sensors, the radar sensor has an advantage in that there is little deterioration in detection performance due to climate change. In addition, in recent automotive radar systems, it is possible to achieve high range resolution by using a wider bandwidth in a frequency-modulated continuous wave (FMCW) radar system [1]. Moreover, high angular resolution can be achieved by using a multiple-input and multiple-output (MIMO) antenna system [2].

In general, a constant false alarm rate (CFAR) algorithm [3] is the most widely used for automatic target detection in the automotive FMCW radar systems. The factors determining the detection performance of the CFAR algorithm include the number of guard cells, the number of training cells, and the probability of false alarm. If these factors are not set properly in the CFAR algorithm, the probabilities of missing the target increase. In addition, it is difficult to efficiently detect targets with fixed factor values because the pattern of the received signal varies depending on the driving environment [4]. Therefore, an improved automatic target detection method is required compared to the conventional CFAR-based method.

To overcome the problems of the conventional CFAR algorithm, studies applying deep learning techniques to the target detection were introduced in [5,6]. In [5], deep learning was applied to the process of estimating the noise level in the conventional CFAR algorithm. In addition, the authors in [6] proposed an artificial neural network to replace the conventional cell averaging (CA)-CFAR algorithm. Moreover, convolutional neural networks (CNNs) [7,8] or U-shaped neural networks (i.e., U-nets) [9,10] were used to detect targets on the range–velocity plane. Recently, deep learning techniques to replace the CFAR algorithm in the automotive MIMO FMCW radar system were also introduced in [11,12]. A U-net-based target detector was proposed in [11] for detecting a vulnerable road user on the range-angle (RA) map. In addition, the authors in [12] compensated for the disadvantages of the conventional CFAR algorithm by replacing the peak detection step of the CFAR algorithm with the deep neural network.

In this paper, we propose a method for detecting targets using an autoencoder (AE) [13], which is one of the deep learning techniques. Recently, the AEs have been actively applied to automotive radar sensor data for various purposes. For example, the AEs were used for suppressing mutual interference between automotive radar systems [14,15,16]. In addition, the authors in [17,18] used the AEs to suppress noise components on the range–velocity plane. However, few studies have been conducted to detect targets on the RA map using AEs. Therefore, we apply the AEs to the target detection result on the RA map to find the significant signal components, which enables efficient target detection. In other words, the entire detection result is compressed on the encoder side, and only significant signal components are restored on the decoder side. To this end, we propose a method for generating an appropriate data set for training AE and determining the structure of the autoencoder.

First, we obtain radar sensor data in the parking lot environment using the automotive MIMO FMCW radar. From the acquired radar sensor data, RA maps indicating the position information of the target are generated. Then, we design the AE consisting of an encoder and a decoder that take the RA maps as input. By changing the number of convolutional layers in the encoder, the number of upsampling layers in the decoder, and the number of filters used in each layer, we determine the AE structure that exhibits a high signal-to-noise ratio (SNR) in the RA map. Finally, the performance of the proposed AE-based target detection method is compared with the detection performances of several types of two-dimensional (2D) CFAR algorithms (e.g., CA-CFAR [19], the order-statistics (OS)-CFAR [19], the greatest of cell averaging (GOCA)-CFAR [20], and the smallest of cell averaging (SOCA)-CFAR [20]). As a performance evaluation measure, the correlation coefficient with the RA map labeled with the actual target position is calculated in each target detection method.

In summary, the main contributions of this study can be summarized as follows:

- Through the proposed AE-based target detection method, meaningful targets can be immediately extracted in the RA map, which can replace the conventional CFAR algorithms.
- The process of setting parameters in the CFAR algorithms (e.g., the number of guard cells, the number of training cells, or the false alarm probability) is not required in the proposed method. Instead, in the AE-based target detection, only retraining needs to be performed based on the determined structure.
- To detect only meaningful targets in a noisy environment, the proposed AE-based detectors require only a small amount of training data sets. If the size of the training data set is large, even the noise component may be reconstructed by the decoder.

The remainder of the paper is organized as follows. In Section 2, we introduce the basic principles for estimating target information in the MIMO FMCW radar system. Then, we describe the radar signal measurement environment and present the target detection result in the environment in Section 3. Next, the AE-based target detection in the MIMO FMCW radar system is proposed in Section 4, and its detection performance is also evaluated in this section. Finally, we conclude this paper in Section 5.

## 2. Target Detection in MIMO FMCW Radar System

### 2.1. Radar Data Cube Generation in MIMO FMCW Radar System

**Figure 1:**
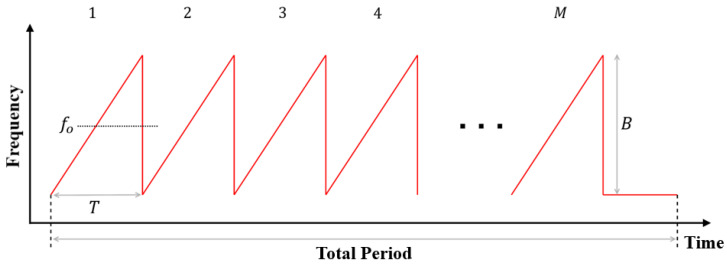
Waveform transmitted from the FMCW radar system.

**Figure 2:**
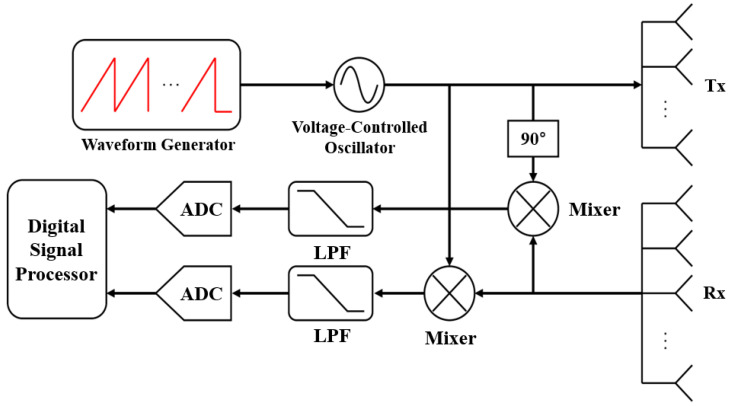
Block diagram of the MIMO FMCW radar system.

As shown in Figure 1, *M* chirps whose frequency increase linearly with time are sequentially transmitted in the FMCW radar system. In the figure, $f_{o}$, *B*, and *T* denote the center frequency, the bandwidth, and the sweep time of each chirp, respectively. Let us assume that the transmitted FMCW radar signal is reflected from the *k*-th target moving at a velocity of $v_{k}$ at a distance of $d_{k}$. Then, the received signal includes a time delay due to $d_{k}$ between the radar and the *k*-th target and the Doppler frequency due to $v_{k}$ between the radar and the *k*-th target. The received signal is down-converted to a baseband signal by passing through a frequency mixer and a low-pass filter (LPF), as shown in Figure 2.

Finally, the signal sampled at the analog-to-digital converter (ADC) can be expressed as

(1) $$\begin{array}{ccc} x[n,\;m] & = & \sum_{k=1}^{K}\alpha_{k}\exp\left(j2\pi \left(\frac{2d_{k}B}{cT}T_{s}n+\frac{2v_{k}f_{o}T}{c}m+\frac{2d_{k}f_{o}}{c}\right)\right), \end{array}$$

where $\alpha_{k}$ denotes the amplitude of the baseband signal and *K* denotes the total number of targets. In addition, *c* indicates the speed of light. In (1), n(n=1,2,…,N) indicates the index for time samples in each chirp and m(m=1,2,…,M) indicates the index for each chirp. In addition, $T_{s}$ represents the time interval between two time samples.

Moreover, to estimate the angle information of targets, we use the uniform linear array antenna system consisting of multiple antenna elements. Assuming that the angle between the *k*-th target and the center of the array antenna is expressed $\theta_{k}$, (1) can be expanded as

(2) $$\begin{array}{c} x\left[n,\;m,\;l\right]=\sum_{k=1}^{K}\alpha_{k}\exp\left(j2\pi \left(\frac{2d_{k}B}{cT}T_{s}n+\frac{2v_{k}f_{o}T}{c}m+\frac{f_{o}d\sin\theta_{k}}{c}\left(l-1\right)+\frac{2d_{k}f_{o}}{c}\right)\right), \end{array}$$

where l(l=1,2,…,L) is the index for the receiving antenna elements and *d* is the spacing between two antenna elements.

**Figure 3:**
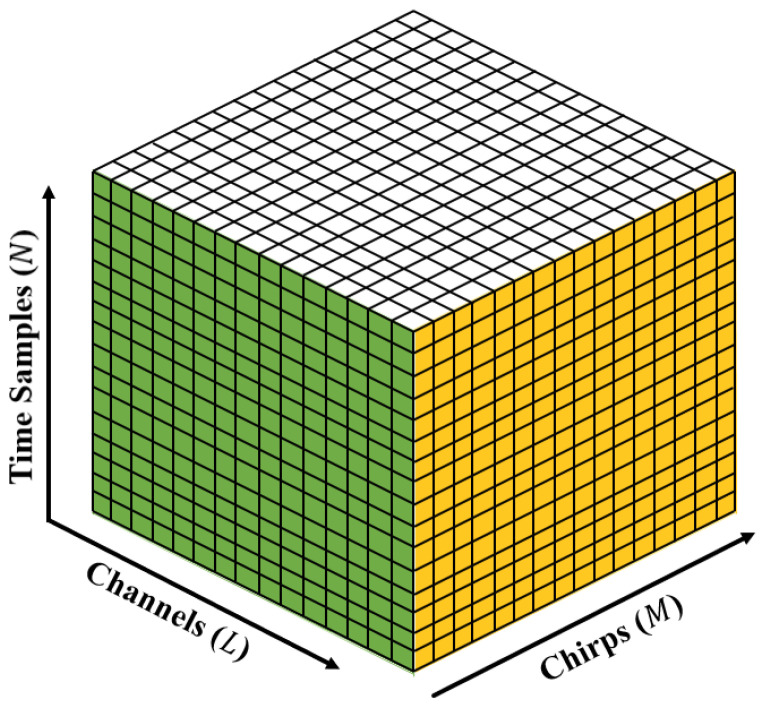
Generated radar data cube in the MIMO FMCW radar system.

In the MIMO antenna system in which the number of transmit antenna elements is $N_{T}$ and the number of receiving antenna elements is $N_{R}$, the total number of receiving channels *L* can be virtually increased to a maximum of $N_{T}\times N_{R}$ [2]. In addition, the phase difference between the virtually generated channels is determined by the distance $d_{T}$ between the transmit antenna elements and the distance $d_{R}$ between the receive antenna elements. In relation to (2), the finally generated N×M×L three-dimensional (3D) radar data is expressed in Figure 3.

### 2.2. Target Information Estimation Using Radar Data Cube

**Figure 4:**
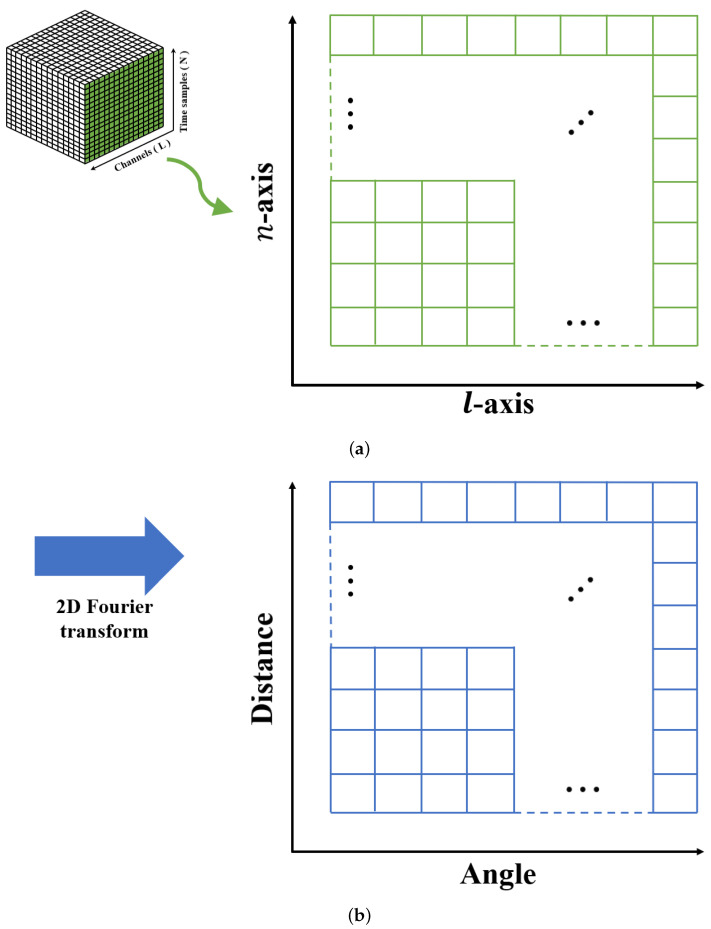
(**a**) Time-sampled baseband signals from all antenna elements and (**b**) the result of applying the 2D Fourier transform to the 2D data matrix.

In general, the distance and angle of the target are the most important in expressing the location information of the target. The method of estimating the distance and angle information of the target in a given radar data cube is as follows. For a fixed $m^{*}$-th chirp, the time-sampled baseband signal of (2) can be represented as a 2D signal matrix, which is shown in Figure 4a. For this matrix, the distance to the target can be obtained by applying the Fourier transform in the direction of the sampling axis (i.e., *n*-axis), and the angle of the target can be extracted by applying the Fourier transform in the direction of the antenna axis (i.e., *l*-axis) [21], as shown in Figure 4b. In other words, the 2D data matrix of Figure 4b is obtained by applying a 2D Fourier transform to the time-sampled signals from all antenna elements for fixed $m^{*}$, which can be expressed as

(3) $$\begin{array}{ccc} X[p,\;r] & = & \frac{1}{NL}\sum_{n=1}^{N}\sum_{l=1}^{L}x\left[n,\;m^{*},\;l\right]\times \exp\left(-j2\pi \left(\frac{p}{N}n+\frac{r}{L}l\right)\right)\\ & & (p=1,\;2,\ldots ,\;P,\;r=1,\;2,\;\ldots ,\;R). \end{array}$$

In (3), *p* and *r* represent indices for the range and the angle in the Fourier transform domain, respectively.

In summary, by applying the 2D Fourier transform to the time-sampled signals from all receiving antenna elements, the distance and angle information of the target can be estimated at the same time. Similarly, if the 2D Fourier transform is applied in the *n*-axis and the *m*-axis directions for fixed $l^{*}$ in the radar data cube, the range and velocity information of the target can be estimated at the same time. In addition, it is possible to simultaneously estimate the velocity and angle information of the target by applying the 2D Fourier transform in the *m*-axis and *l*-axis directions for the fixed $n^{*}$.

## 3. Radar Signal Measurement and Target Detection Result

In the experiment, we used the MIMO FMCW radar sensor (i.e., AWR2243 [22]) manufactured by Texas Instruments. The radar sensor uses 78.3 GHz and 2.53 GHz as the center frequency and the bandwidth, respectively. In addition, 64 chirps were used and 256 time samples were obtained from each chirp. Moreover, the number of transmit antenna elements and the number of receiving antenna elements were 12 and 16, respectively. According to the MIMO antenna principle, a total of 192 (i.e., $N_{t}\times N_{R}=192$) virtual receiving channels can be generated, but in the case of the AWR2243, only 86 channels exist in the azimuth direction. Thus, we use all the signals received on those 86 channels (i.e., L=86). The specifications of the radar system we used are summarized in Table 1.

**Figure 5:**
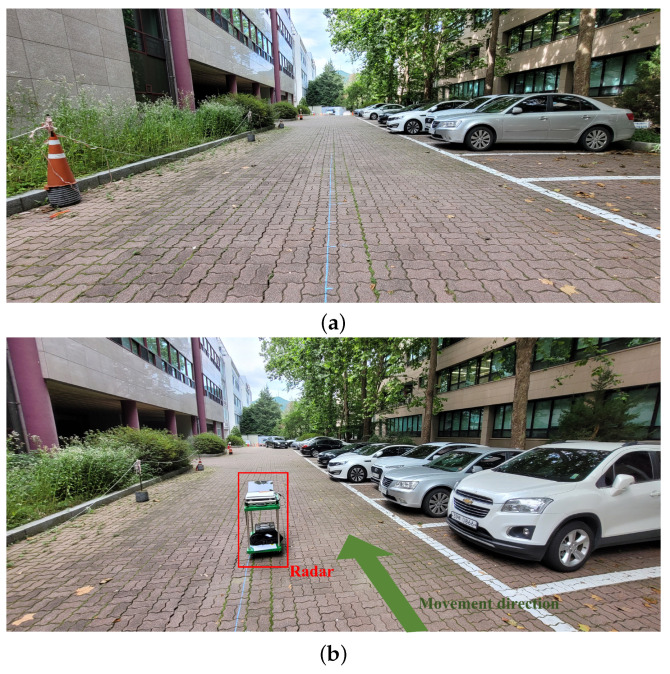
(**a**) Parking lot where the radar signal measurements were conducted and (**b**) the placement of the radar.

**Figure 6:**
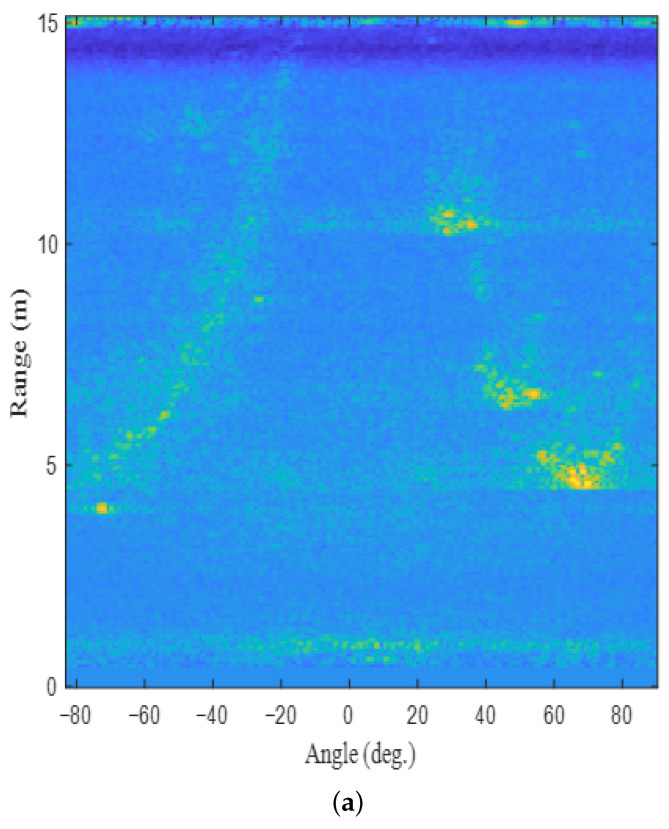

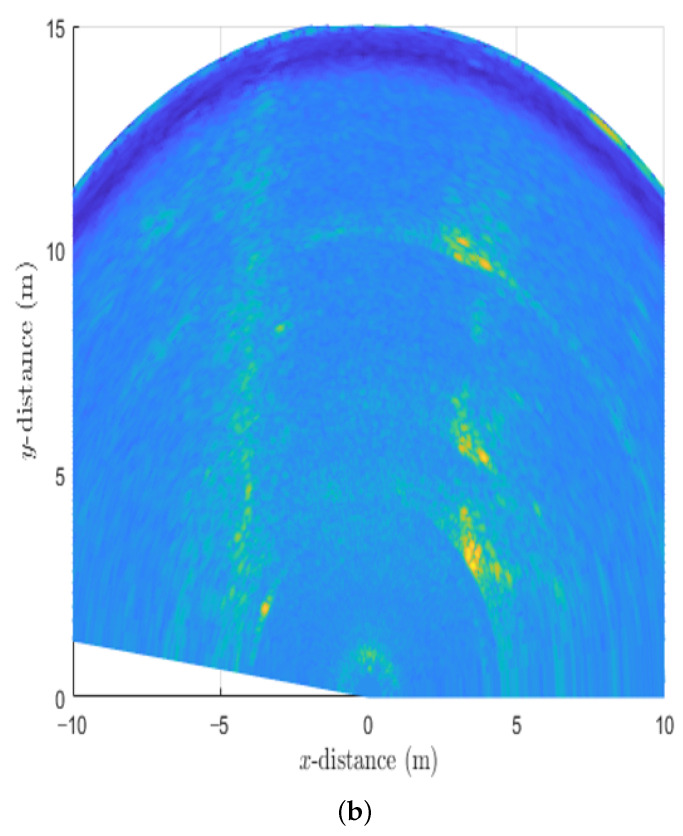
Target detection results: (**a**) on the RA map and (**b**) on the xy plane.

Using this radar system, signal measurements were conducted in the parking lot with cars, as shown in Figure 5a. In this environment, we acquired radar sensor data while moving the cart on which the radar was installed, as shown in Figure 5b. Figure 6 shows the target detection result in the environment of Figure 5. The absolute value of the signal of (3) for the first chirp is shown in Figure 6a. In addition, Figure 6b presents the result of converting the target detection result on the RA map to the distance axes in the *x*-axis and *y*-axis directions. In the figure, the positions that strongly reflect the radar signal are expressed in bright colors (e.g., points marked in yellow). As shown in the figure, the radar signal is strongly reflected from the vehicles parked on the right.

**Figure 7:**
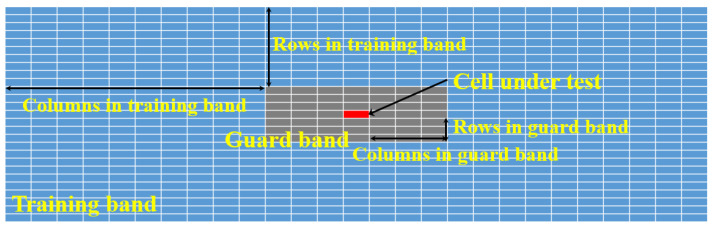
Parameters in the 2D CFAR algorithms.

**Figure 8:**
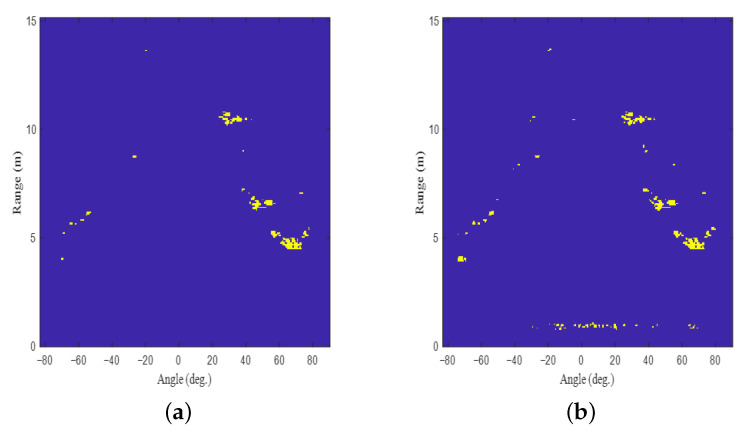

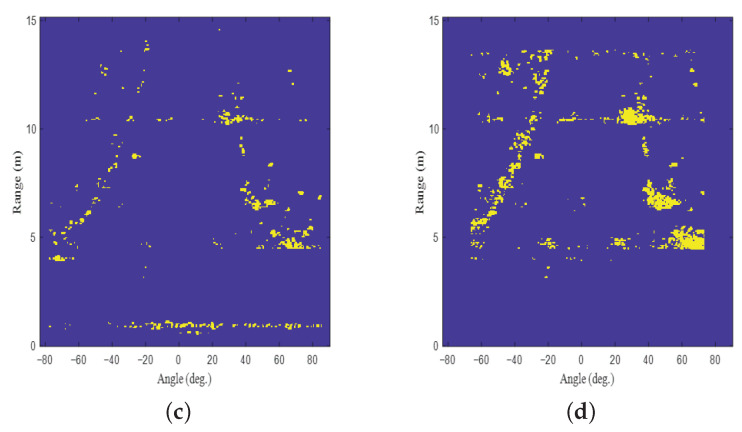
Target detection results when the CA-CFAR algorithm with different parameter values are applied: (**a**) $P_{fa}=0.343$, guard band size: [3×3], training band size: [15×15], (**b**) $P_{fa}=0.356$, guard band size: [2×2], training band size: [5×5], (**c**) $P_{fa}=0.347$, guard band size: [3×3], training band size: [10×10], and (**d**) $P_{fa}=0.358$, guard band size: [5×5], training band size: [20×20].

To extract information about significant targets from the target detection result in Figure 6, a peak detection algorithm must be applied. Conventionally, the CFAR algorithms are widely used to extract points that corresponds to the significant targets in a noisy environment. However, if the number of guard cells, the number of training cells, and the probability of false alarm are set improperly in the 2D CFAR algorithm, the target detection performance is severely degraded. Regarding the 2D CFAR algorithm, the regions corresponding to the guard band size and the training band size are shown in Figure 7. For example, Figure 8 shows the results when the CA-CFAR algorithm with different parameter values are applied to the target detection result in Figure 6. As shown in the figure, the target detection performance depends on factors, such as the probability of false alarm ($P_{fa}$), the guard band size, and the training band size. Thus, there is a need for a target detection method that does not greatly depend on these parameter values.

## 4. Proposed AE-Based Target Detection in RA Map

### 4.1. Structure of AE-Based Target Detector

In this section, we propose to apply the CNN-based AE to extract targets on the RA map. The AE is one of the representative unsupervised learning-based machine learning techniques, and it has the characteristics of manifold learning and a generative model [23]. In general, the AE consists of an encoder that compresses input data and a decoder that reconstructs the data. If we appropriately design the encoder and decoder, only the main signal components can be compressed through the encoder, and they can be reconstructed through the decoder.

**Figure 9:**
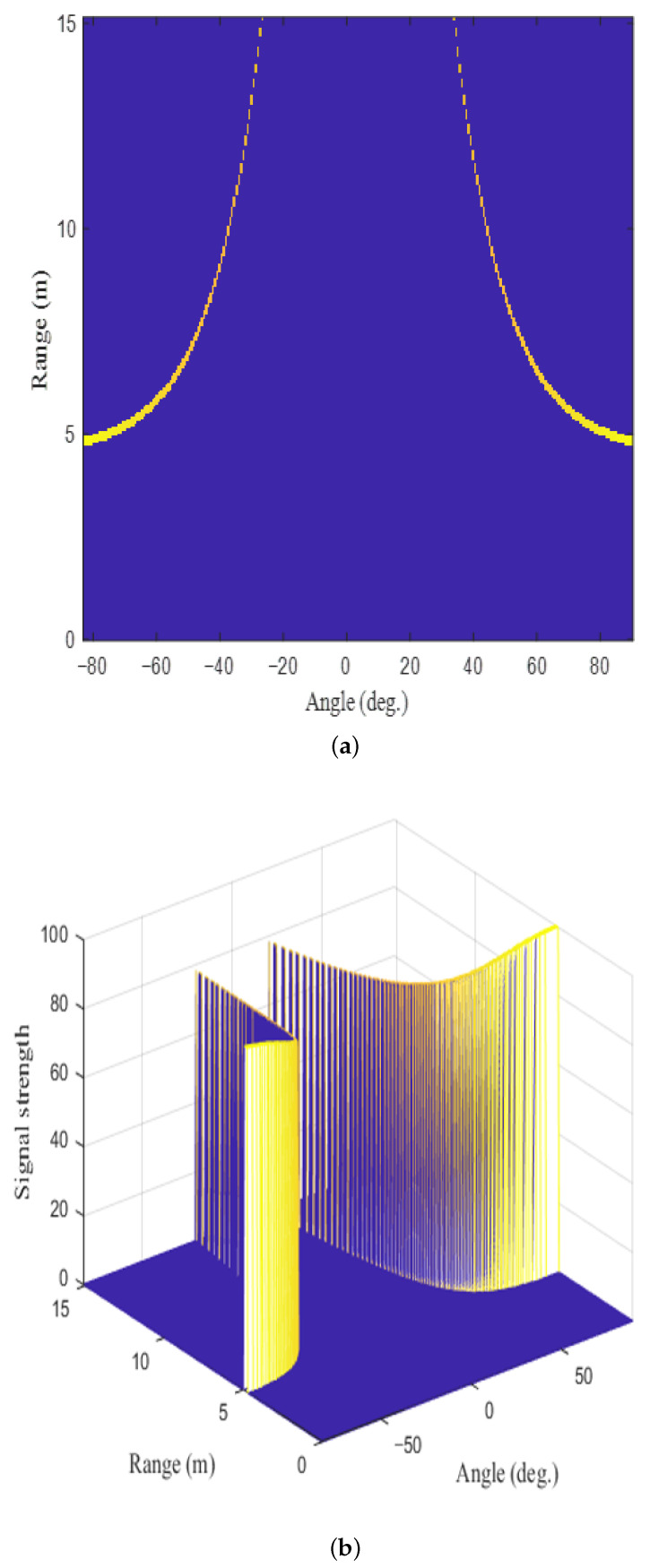
Example of training data: (**a**) a target detection result on the RA map and (**b**) its signal strength.

As shown in Figure 6, dominant stationary targets exist along the left and right parabolas on the RA map. In general, stationary targets appear in the form of parabolas in the automotive MIMO FMCW radar system [24]. Therefore, we decided to design the AE that extracts targets located along two parabolas. First, the input data used for training were generated based on the detection results from the actual radar sensor. For example, one of the training data we generated is shown in Figure 9. Figure 9a,b show the generated target detection result on the RA map and its signal strength, respectively. Because the curvature of the curves is determined by the width of the road on the RA map, we generated parabolas with various curvatures. In addition, the received signal strength varies according to the distance between the targets and the radar. Thus, the received signal strength of each point was established based on the radar equation [25], and it was also readjusted according to the actual signal strength value acquired from the radar system we used. Moreover, because the curve does not form a perfect shape in the actual target detection result, the training data were forcibly distorted to generate inputs such as broken curves. Using this training data set, only strong signal components reflected by the targets are trained. In other words, the encoder is trained to compress only information about signals with high signal strength.

Finally, a total of 200 input data were generated, of which 50% had the form of a perfect parabola, and the remaining 50% had the form of an incomplete parabola. After that, the white Gaussian noise was added to each input data, and the SNR values were set variously in consideration of the actual measurement environment. If the number of training data is large, the encoder and the decoder can overfit to the given data. Thus, it is important to set the number of input data appropriately [26]. If the size of the training data set is large, even the noise component can be reconstructed by the decoder. For the entire data set, 90%, 5%, and 5% were used as a training, validation, and test sets, respectively.

With this data set, we determine the CNN-based AE structure suitable for target detection. In this process, the structures of the encoder and the decoder are designed to be symmetrical to each other to obtain reconstructed results having the same size as the input data size. In addition, one hidden layer on the encoder side consists of a convolution layer and a rectified linear unit (ReLU) layer. On the other hand, one hidden layer on the decoder side includes an upsampling layer and a ReLU layer.

**Figure 10:**
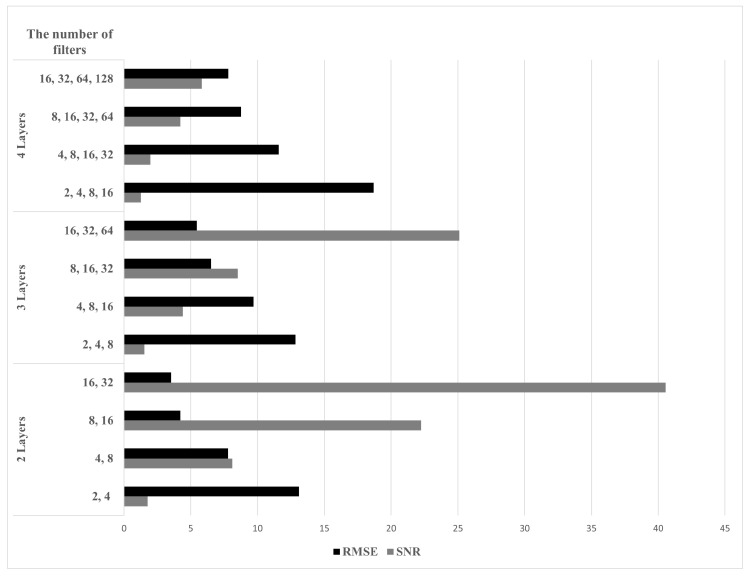
RMSE and SNR values according to the number of hidden layers and the number of filters used in each layer.

Then, by adjusting the number of hidden layers and filters used in the encoder and the decoder, respectively, we evaluated the root mean square error (RMSE) and SNR values to determine the structure of the AE suitable for our data set. The RMSE is calculated as

(4) $$\begin{array}{c} \mathrm{RMSE}=\sqrt{\frac{\sum_{p=1}^{P}\sum_{r=1}^{R}{\left(T_{in}\left[p,\;r\right]-T_{out}\left[p,\;r\right]\right)}^{2}}{PR}}, \end{array}$$

where $T_{in}$ and $T_{out}$ indicate the input and the corresponding output of the designed AE in the validation set, respectively. In addition, *P* and *R* represent the total number of *p* and *r* in the Fourier tranform domain, respectively. By calculating the RMSE value for the validation data set not used for training, it is possible to determine how well the AE structure is trained [27]. In addition, the SNR value was calculated as

(5) $$\begin{array}{c} \gamma =\frac{{\left(\sum_{p=1}^{P}\sum_{r=1}^{R}{\left(I_{target}⨀T_{out}\right)}_{pr}\right)}^{2}}{{\left(\sum_{p=1}^{P}\sum_{r=1}^{R}{\left(I_{noise}⨀T_{out}\right)}_{pr}\right)}^{2}}, \end{array}$$

where $I_{target}$ is the data labeled 1 at the actual target position, and $I_{noise}$ is the data labeled 1 at locations other than the target position. In addition, ⨀ stands for the element-wise multiplication (i.e., Hadamard product). A large value of γ in (5) means that the desired signal component is preserved and many other noise components are suppressed. Figure 10 shows the RMSE and the SNR values according to the number of hidden layers and the number of filters used in each layer. The smaller the RMSE and the higher the SNR, the more suitable the structure is for the target detection.

Moreover, we also calculated the 2D correlation coefficient [28], which is defined as

(6) $$\begin{array}{ccc} \rho & = & \frac{\sum_{p=1}^{P}\sum_{r=1}^{R}\left(I_{target}\left[p,\;r\right]-\frac{\sum_{p=1}^{P}\sum_{r=1}^{R}\left(I_{target}\left[p,\;r\right]\right)}{PR}\right)}{\sqrt{\sum_{p=1}^{P}\sum_{r=1}^{R}{\left(I_{target}\left[p,\;r\right]-\frac{\sum_{p=1}^{P}\sum_{r=1}^{R}\left(I_{target}\left[p,\;r\right]\right)}{PR}\right)}^{2}}}\\ \; & \; & \times \frac{\sum_{p=1}^{P}\sum_{r=1}^{R}\left(T_{out}\left[p,\;r\right]-\frac{\sum_{p=1}^{P}\sum_{r=1}^{R}\left(T_{out}\left[p,\;r\right]\right)}{PR}\right)}{\sqrt{\sum_{p=1}^{P}\sum_{r=1}^{R}{\left(T_{out}\left[p,\;r\right]-\frac{\sum_{p=1}^{P}\sum_{r=1}^{R}\left(T_{out}\left[p,\;r\right]\right)}{PR}\right)}^{2}}}. \end{array}$$

**Figure 11:**
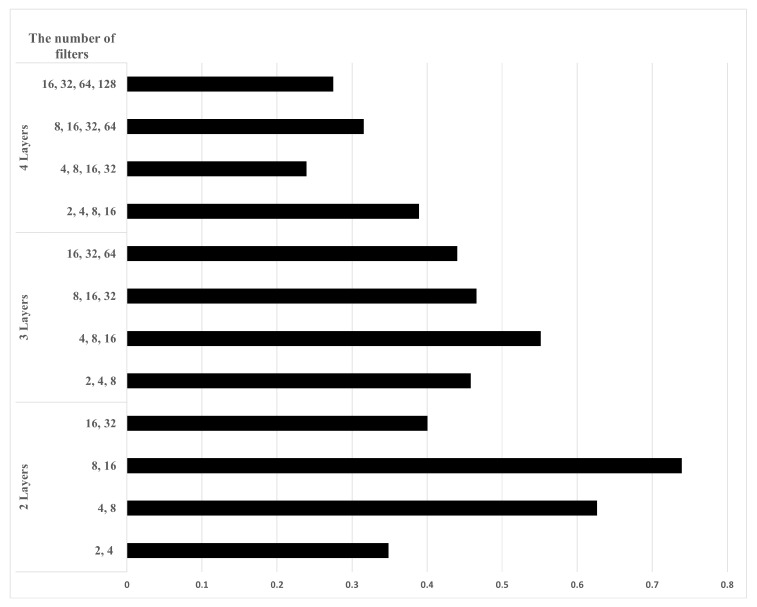
Correlation coefficient values according to the number of hidden layers and the number of filters used in each layer.

In other words, the position of the actual target is compared with the position of the target in the output of the proposed AE. A high correlation coefficient means that the final target detection result extracted through the proposed AE is highly similar to the RA map containing the actual target location. Figure 11 shows the correlation coefficient values according to the number of hidden layers and the number of filters used in each layer. From the RMSE, SNR, and correlation coefficient values, using two hidden layers each for the encoder and the decoder shows the best performance. In addition, the AE structure using eight and 16 filters in the first and the second hidden layers, respectively, gives the best performance.

**Figure 12:**
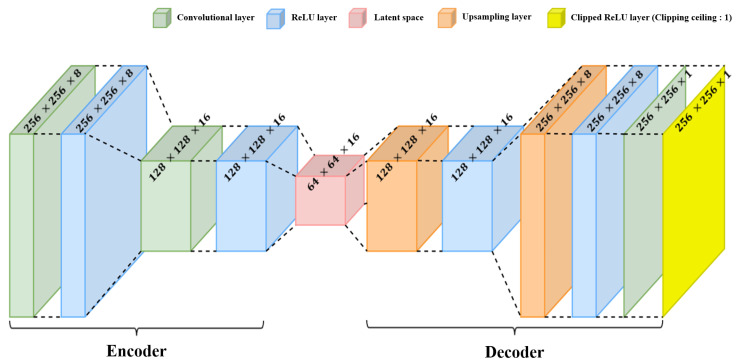
Structure of the proposed AE-based target detector.

Finally, Figure 12 shows the structure of the determined CNN-based AE. To extract the features from the RA map, a total of two executions are performed with a set of the convolutional layer, the ReLU layer, and the max pooling layer on the encoder part. In addition, the decoder restores the data using the upsampling layer, the ReLU layer, and the clipped ReLU layer. The clipped ReLU layer forces the output value to be between 0 and 1 to prevent the output value from becoming too large.

### 4.2. Performance Evaluation

**Figure 13:**
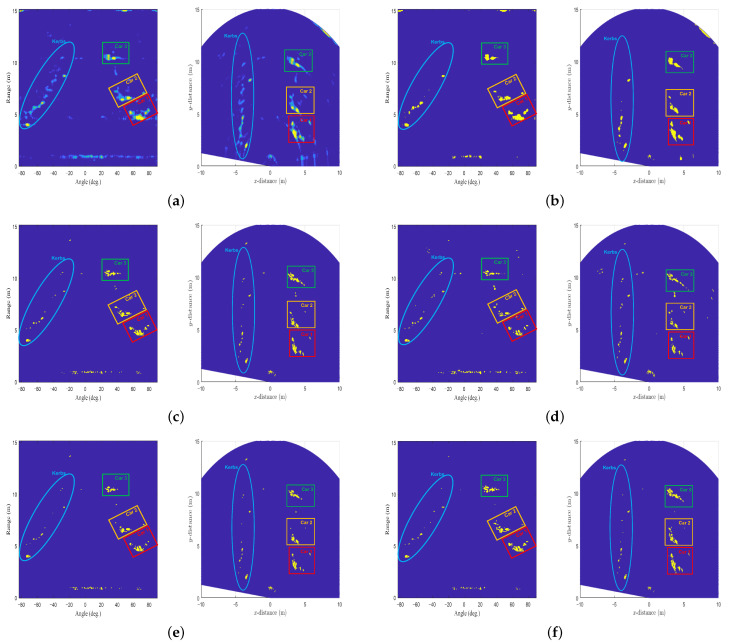
Final target detection results: (**a**) the proposed AE-based target detector, (**b**) the proposed AE-based target detector (binarized), (**c**) the CA-CFAR, (**d**) the OS-CFAR, (**e**) the GOCA-CFAR, and (**f**) the SOCA-CFAR algorithms.

To evaluate the performance of the AE-based target detector, the detection results of applying the CA-CFAR, OS-CFAR, GOCA-CFAR, and SOCA-CFAR algorithms were compared with that of the proposed method. As already mentioned, the performance of the CFAR algorithm depends on the values of parameters, such as the false alarm probability, the guard band size, and the training band size. In the performance evaluation, those parameters in each CFAR algorithm were empirically determined to provide appropriate detection performance, and their values are summarized in Table 2. The corresponding detection results including the proposed method are shown in Figure 13. If the detection result of Figure 6 is passed through the structure of Figure 12, Figure 13a is immediately generated. One of the advantages of the AE-based target detector is that the signal strength information is preserved when compared with the detection results in the CFAR algorithm.

Table 2 also shows the correlation coefficient values in (6) for the proposed AE-based target detector and the conventional CFAR algorithms. To evaluate the statistical performance of the proposed method, dozens of measurements were performed, and the correlation coefficient values were averaged. First, the predicted output of the AE was compared to the data labeled with the location of the actual target. Then, similar to the results of the CFAR algoruthms, the predicted output was also binarized to calculate the correlation coefficient value. As shown in the table, the AE output has a high degree of similarity to the data labeled with the actual target location.

**Figure 14:**
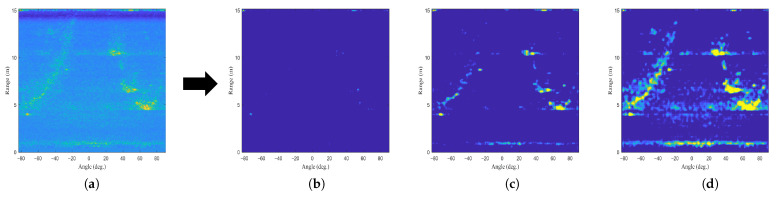
AE outputs according to the number of training data: (**a**) the RA map before passing the AE, (**b**) the AE output when the number of the training data is 100, (**c**) the AE output when the number of the training data is 200, (**d**) and the AE output when the number of the training data is 300.

Moreover, we verify how the performance of AE changes according to the number of training data. Figure 14 shows the outputs when the AE is trained with 100, 200, and 300 training data. If the number of training data is 100, the strong signal component is not well trained (i.e., Figure 14b), and if the number is 300, the noise component is also trained (i.e., Figure 14d). Therefore, it is important to set the number of training data appropriately for the performance of the AE.

**Figure 15:**
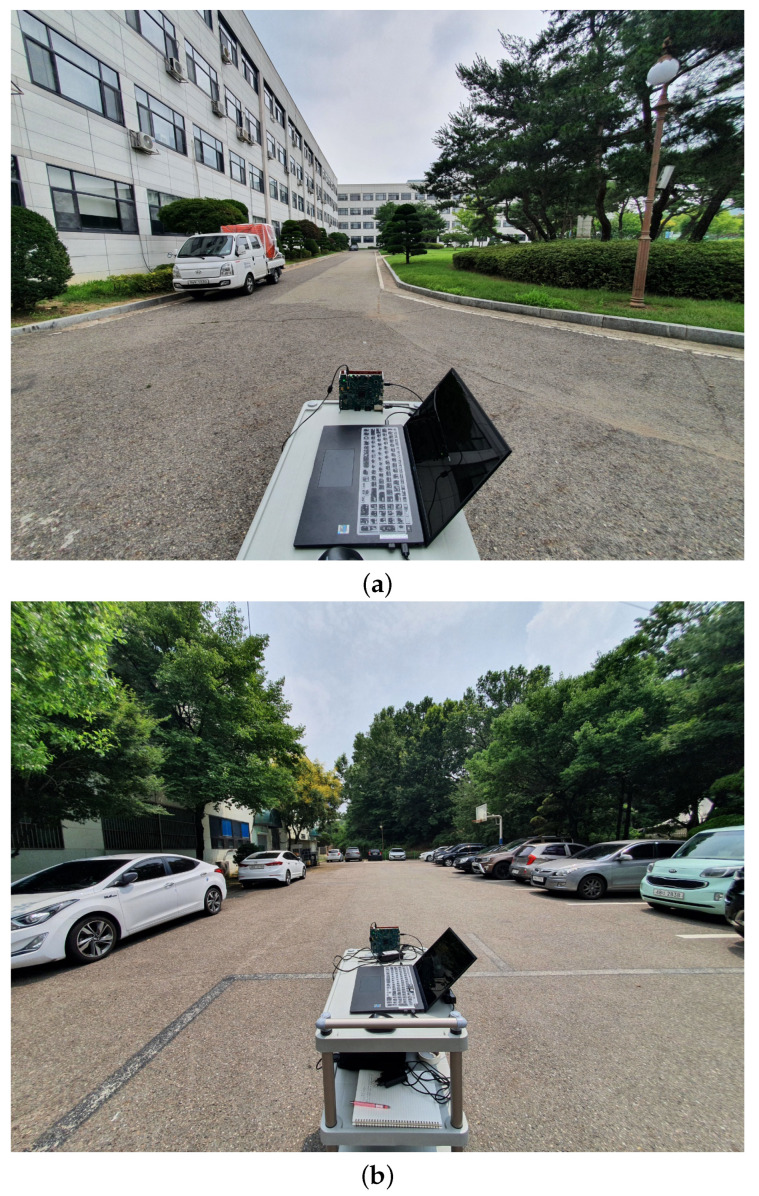
Environments in which radar signal measurements were performed: (**a**) the road in front of the building and (**b**) a parking lot with several vehicles.

**Figure 16:**
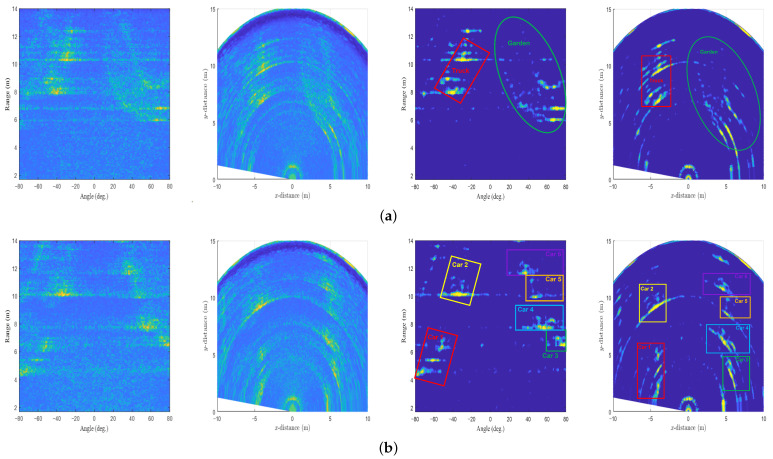
Target detection results in different environments: (**a**) the road in front of the building and (**b**) a parking lot with several vehicles.

Finally, we conducted additional experiments to evaluate the performance of the proposed AE-based target detector. The experiments were conducted in other outdoor environments, which are shown in Figure 15. As shown in Figure 16, even if the AE trained from the radar sensor data acquired in the environment of Figure 5 is applied to the sensor data acquired in the environment of Figure 15, the target detection performance is guaranteed to some extent.

## 5. Conclusions

In the MIMO FMCW radar system, the position information of the target can be immediately estimated from the RA map. To extract significant signal components corresponding to targets from the RA map, we proposed the AE-based target detection method. We designed a CNN-based AE with the RA maps as input by changing the number of hidden layers and the number of filters used in each layer. After completing the training process, the trained AE immediately locates the target on the RA map, ensuring high SNR values. Finally, to verify the effectiveness of the proposed method, the correlation between the target detection result and the data labeled with the actual target location was measured. The proposed AE-based target detector had a correlation coefficient value of 0.7396 on average, which is high compared to the conventional CFAR-based target detection methods.

## Figures and Tables

**Table 1:** Specifications of the MIMO FMCW radar system.

Parameter	Value
Center frequency, $f_{o}$	78.3 GHz
Operating bandwidth, *B*	2.53 GHz
Sweep time, *T*	46 μs
Sampling frequency, $f_{s}$	8 MHz
Sampling interval, $T_{s}$	125 ns
The number of chirps, *M*	64
The number of time samples in each chirp, *N*	256
The number of transmit antenna elements, $N_{t}$	12
The number of receiving antenna elements, $N_{r}$	16
The number of virtual receiving channels, *L*	86

**Table 2:** Correlation coefficient values for the proposed AE-based target detector and the conventional CFAR algorithms.

Method	$P_{\mathrm{fa}}$	Training Band Size	Guard Band Size	ρ
AE-based target detector	N/A	N/A	N/A	0.7396
AE-based target detector (binarized)	N/A	N/A	N/A	0.7020
CA-CFAR algorithm	0.347	[10×10]	[3×3]	0.6994
OS-CFAR algorithm	0.237	[10×10]	[2×2]	0.6012
GOCA-CFAR algorithm	0.336	[10×10]	[3×3]	0.6547
SOCA-CFAR algorithm	0.356	[10×10]	[3×3]	0.7256

## Data Availability

Data sharing not applicable.

## Abbreviations

The following abbreviations are used in this manuscript:

2D	Two-dimensional
3D	Three-dimensional
ADC	Analog-to-digital converter
AE	Autoencoder
CA	Cell averaging
CFAR	Constant false alarm rate
CNN	Convolutional neural network
FMCW	Frequency-modulated continuous wave
GOCA	Greatest of cell averaging
LPF	Low-pass filter
MIMO	Multiple-input and multiple-output
RA	Range angle
RMSE	Root mean square error
SNR	Signal-to-noise ratio
SOCA	Smallest of cell averaging
